# Risk of bladder cancer in patients with diabetes: a retrospective cohort study

**DOI:** 10.1186/2049-3258-73-S1-P4

**Published:** 2015-09-17

**Authors:** Maria (Mieke) Goossens, Maurice Zeegers, Marloes Bazelier, Marie De Bruin, Frank Buntinx, Frank De Vries

**Affiliations:** 1 KU Leuven, Leuven, Belgium; 2 University Maastricht, Maastricht, Netherlands; 3 Utrecht University, Utrecht, Netherlands

## Objective

The objective of this study was to examine the association between diabetes and both urinary bladder cancer (UBC) risk and mortality.

## Methods

We conducted a retrospective cohort study using data from the UK Clinical Practice Research Datalink (CPRD) linked to the Office of National Statistics (ONS). Patients diagnosed with diabetes mellitus type 1 or 2 or using anti-diabetic drugs (ADD) were compared to matched non-diabetic controls. Cox proportional hazards models were used to estimate the risk and mortality of UBC. We adjusted for age, sex, smoking status and BMI.

## Results

The cohort included 329,168 patients using ADD and 307,315 controls with 1,295 and 1,071 patients, respectively, diagnosed as having UBC during follow-up. The adjusted hazard ratios (HR) of UBC were 0.77 (95% CI 0.57-1.05) and 1.04 (95% CI 0.96-1.14) for type 1 and 2 diabetes, respectively. These results were similar if we restricted our analysis to an inception cohort. We noticed a small increased risk during the first year after diagnosis (HR = 1.26 (95% CI 1.05-1.52)), which could be explained by detection bias. There was no influence of the severity of diabetes as measured by the HbA1c. Mortality of UBC was not increased for patients with either type 1 (HR = 0.95 (95% CI 0.39-2.34)) or type 2 diabetes (HR = 1.16 (95% CI 0.91-1.46)).

## Conclusion

Neither the risk of UBC, nor the mortality from UBC, was increased in type 1 and patients with type 2 diabetes in the CPRD data.

